# Synergistic *Artemisia monosperma* with royal jelly: antibacterial, antioxidant, antibiofilm, and anti-Alzheimer assay

**DOI:** 10.1186/s13568-025-01838-0

**Published:** 2025-03-13

**Authors:** Naglaa Elshafey, Sally Said Ehmedan, Nashwa Hagagy, Shereen M. Elbanna, Reham Z. Sadek

**Affiliations:** 1 Botany and Microbiology Department, Faculty of Science, Arish University, Al-Arish, 45511 Egypt; 2 Department of Biology, College of Science & Arts at Khulis, University of Jeddah, 21959 Jeddah, Saudi Arabia; 3 Botany and Microbiology Department, Faculty of Science, Suez Canal University, Ismailia, 41522 Egypt; 4 Zoology Department, Faculty of Science, Suez Canal University, Ismailia, 41522 Egypt

**Keywords:** *Artemisia monosperma*, Anti Alzheimer assay, Royal jelly, Antibiofilm, Antibacterial activity, Molecular docking

## Abstract

This investigation aimed to examine the synergistic effects of fresh royal jelly and local wild *Artemisia monosperma* leaf extract as antibacterial, antioxidant, antibiofilm, and anti-Alzheimer activity. Gas chromatography-mass spectrometry (GC–MS) identified 16 compounds in *Artemisia monosperma*, including tricosadiynoic acid, hexadecenoic acid, octadecenoic acid. In contrast, fresh royal jelly contained 13 compounds, including dodecanoic acid, octadecynoic acid, hexadecenoic acid, heptatriacotanol, and their derivatives. The *Artemisia monosperma* extract exhibited significant antioxidant activity in the DPPH assay, with IC_50_ value of 5.48 ± 0.002 µg/mL. Fresh royal jelly exhibited an IC_50_ value of 14.56 ± 0.004 µg/mL. Both substances exhibited significant antibacterial activity in comparison to gentamycin. The Synergistic combination (1:1) effectively suppressed the growth of multidrug-resistant bacterial species, including *Bacillus subtilis* (ATCC 6633), *Enterococcus faecalis* (ATCC 10541), *Staphylococcus aureus* (ATCC 6538), *Klebsiella pneumoniae* (ATCC 13883), *Salmonella typhi* (ATCC 6539), and *Pseudomonas aeruginosa* (ATCC 90274), and decreased biofilm activity. Additionally, in vitro the of inhibition activity (IC_50_) of the Butyrylcholinesterase enzyme (BChE) for the plant extract, royal jelly and the Synergistic combination were 4.35 ± 0.002 µg/mL, 4.9 ± 0.002 µg/mL, and 3.55 ± 0.002 µg/mL, respectively while the IC_50_ of rivastigmine (positive control) was 3.9 ± 0.002 µg/mL. in silico analysis reported that the bioactive compounds demonstrated high binding affinities, between − 6.6 and − 10.3 kcal/mol, against the human acetylcholinesterase protein, beside ADMET analysis exhibited high gastrointestinal absorption and potential inhibitory effects on CYP1A2 and CYP2C9 enzymes. Our study indicated that the synergistic effect of *Artemisia monosperma* and royal jelly bioactive compounds exhibited a promising antibacterial, antioxidant, antibiofilm, and acetylcholinesterase inhibitory activities.

**Supplementary Information:**

The online version contains supplementary material available at 10.1186/s13568-025-01838-0.

## Introduction

Egypt Mediterranean coastal land located in a region characterized by a dry and arid climate, Plant species in these habitats have evolved different strategies to adapt to stressful abiotic and biotic factors. These strategies include changes in morphological and anatomical structures, the development of chemical defense mechanisms, and increased antioxidant activity (Abdelaal et al. 2018, 2019). Plants are abundant in secondary metabolites, which are known for their pharmacological and protective properties (Eswaraiah et al. 2020; Mohammed et al. 2022)**.***Artemisia monosperma*, a member of the Asteraceae family, is a flowering species within the *Artemisia* genus. It is characterized by its dense foliage and slender, elongated leaves, and plays a significant role in ecological succession by helping to stabilize sand dunes (El-Sherbeny et al. 2021). *Artemisia monosperma* is prevalent in the desert plains and valleys (wadis) of the Arabian Peninsula, and its leaves are extensively utilized in traditional medicine in Egypt (Guetat et al. 2017). Also known as Wormwood or Al-Ader’ in Egypt, this aromatic perennial shrub is native to Egypt and is widespread across various Arabic countries (Hussain et al. 2017). The plant is also used by Bedouins in the Sinai Peninsula for treating diabetes, relieving spasms, and alleviating colds and pain (Mostafa et al. 2018). Similarly, royal jelly, produced by young worker bees in honeybee colonies, has antioxidant, antimicrobial, antiallergic, neuroprotective, antihypertensive, anti-diarrheal, and effects on enzyme inhibitors and conditions like Alzheimer's, depression, infertility, digestive disorders, anemia, and stress-related illnesses (Bouamama et al. 2021). Also, contains phenolic compounds as flavonoids and polyphenols that could neutralize free radicals and reduce oxidative stress which have antioxidant properties contributing to the jelly’s health benefits and medical uses (Oršolić and Jazvinšćak Jembrek 2024). In the present study, *Artemisia monosperma* and Royal jelly were selected due to their medicinal properties, but they differ significantly in their chemical compositions, these differences highlight the diverse potential applications in traditional and modern medicine, and the synergistic effect between both Suggesting a promising future for its inclusion in various therapeutic regimens.

## Materials and methods

### Plants and royal jelly collection

**Fig. 1:**
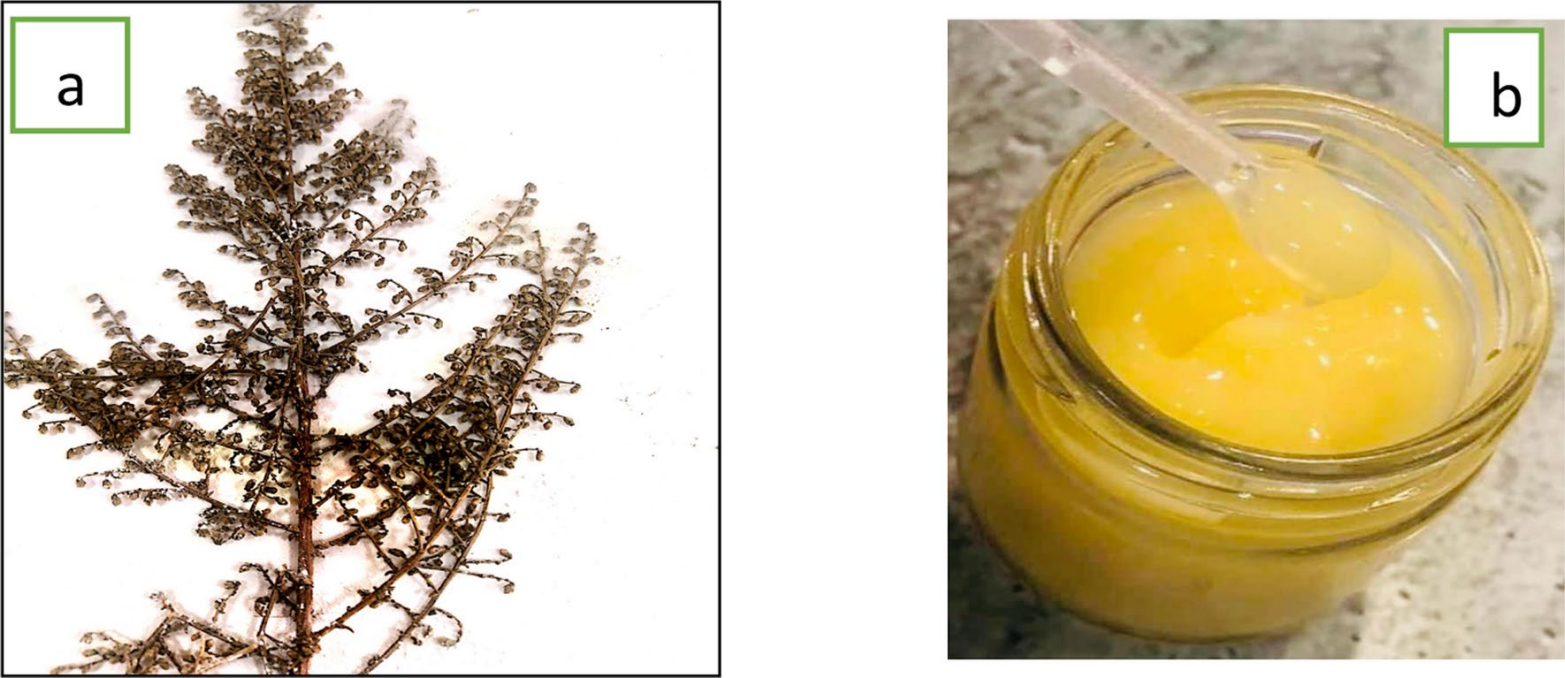
The collected samples **a*** Artemisia monosperma*** b** fresh Royal Jelly sample

*Artemisia monosperma* was collected from north Sinai governorate, Egypt. And identified by plant taxonomist, botany department, Arish university. The voucher specimens with accession number A3448 (Fig. 1a) and fresh Royal jelly samples (Fig. 1b) were freshly harvested and collected from queen cells in beehives. The collection process involves carefully removing the queen larvae and then extracting the royal jelly from the cells, with a small spatula or suction device into a 25 mL glass bottle then transported to the laboratory in an icebox approximately 4 °C (Sabatini et al. 2009).

### Extraction of bioactive compounds from *Artemisia monosperma*

The ethanol extract (70%) was obtained by conducting a thorough cold maceration of the dried leaves (5 gm/100 ml). Under reduced pressure the solvent was evaporated at 40 °C. After drying, the byproducts are stored for additional chemical and biological analysis.

### Gas chromatography and mass spectrometry (GC/MS) analysis

The chemical composition of *Artemisia monosperma* and crude royal jelly was assayed using a Trace GC1310-ISQ mass spectrometer from Thermo Scientific in Austin, TX, USA. The direct capillary column TG-5MS measured 30 m in length, 0.25 mm in diameter, and 0.25 µm in thickness. The temperature in the column oven rose from 50 to 230 °C for two minutes at a rate of 5 °C per minute. The carrier gas was helium, flowing at 1 ml/min, while the injector and MS transfer line were consistently maintained at 250 °C and 260 °C, respectively, during the experiment. Following a three-minute solvent delay, 1 ml of diluted samples were injected into a gas chromatograph using an AS1300 autosampler in split mode. Full-scan electron impact mass spectra from m/z 40 to 1000 were acquired at ionization voltages of 70 eV. The temperature of the ion source reached to 200 °C. Mass spectra and retention times were analyzed against the WILEY 09 and NIST 11 mass spectral databases, facilitating the identification of components.

### FTIR spectroscopy measurement

To identify the most important functional groups in samples were prepared by grinding 1 mg of dried *Artemisia monosperma* and crud royal jelly separately with 100 mg of KBr and pressed at 7500 kg pressure with hydraulic press to acquire transparent pellet. Pellets for each royal jelly and *Artemisia monosperma* were made by the same procedure and analyzed with FTIR spectrometer at the range of 500–4000 cm^−1^ and 400–4000 cm^−1^ respectively (Ezazi et al. 2021).

### Assessment of antioxidant activity via 2,2-diphenyl-1-picrylhydrazyl (DPPH) radical scavenging technique

The free radical scavenging activity of *Artemisia monosperma* and crude Royal Jelly was determined by using 2,2-diphenyl-1-picrylhydrazyl (DPPH). A 0.1 mM DPPH solution in ethanol was prepared. 1 ml of this solution was mixed with 3 ml of various substances in ethanol at different concentrations (3.9, 7.8, 15.62, 31.25, 62.5, 125, 250, 500, 1000 μg/ml). Samples soluble in ethanol were employed, with varying concentrations produced through a dilution procedure. The mixture was vigorously agitated and allowed to rest at an ambient temperature for 30 min. Absorbance was measured at 517 nm using a spectrophotometer (UV–VIS Milton Roy). The experiment was performed in triplicate, utilizing ascorbic acid as the reference standard component (González-Palma et al. 2016). The IC_50_ value of the sample, indicative of the concentration required to inhibit 50% of the 2,2-diphenyl-1-picrylhydrazyl (DPPH) free radical, was established through a logarithmic dose-inhibition curve analysis. A reduced absorbance of the reaction mixture related to increased free radical activity. The 2,2-diphenyl-1-picrylhydrazyl (DPPH) scavenging effect percentage was calculated using the equation provided by Shekhar and Anju (2014).

$$ {\text{DPPH scavenging effect }}\left( \% \right) \, = {\text{ A}}_{0} - {\text{ A}}_{{1}} /{\text{ A}}_{0} \times { 1}00. $$

where A_0_ was the absorbance of control reaction and A_1_ was the absorbance in the presence of test or standard sample.

### Antibacterial activity and determination of MIC and MBC concentration

The antibacterial activity of the *Artemisia monosperma*, royal jelly bioactive compounds and their 1:1 combination, was evaluated against multidrug-resistant strains from Ain Shams Hospital, as *Bacillus subtilis* (ATCC 6633), *Enterococcus faecalis* (ATCC 10541), *Staphylococcus aureus* (ATCC 6538), *Klebsiella pneumoniae* (ATCC 13883), *Salmonella typhi* (ATCC 6539), and *Pseudomonas aeruginosa* (ATCC 90274). by using agar diffusion methods. One milliliter of bacterial inoculum was uniformly applied across the entire surface of the agar using a sterile cotton swab (Hamedo et al. 2023). The bioactive compounds were then introduced into a well created with a sterile cork borer measuring 6 mm in diameter. The plates were subsequently incubated at 37 °C. The diameters of the inhibition zone were measured in millimeters relative to gentamycin (30 g/mL), which served as the positive control (Rahman et al. 2011; Hamedo et al. 2023).

The microdilution method was used to evaluate the minimum inhibitory concentration (MIC) and minimum bactericidal concentration (MBC). The MIC for the bioactive substances from royal jelly, *Artemisia monosperma*, and their combination (1:1) was determined using serial dilution in broth media with 1 mL of extract (100 g/mL in DMSO). 10 μL of a bacterial suspension at 5 × 10^6 CFU/mL was added to each test sample and growth control, which consisted of broth and DMSO without the antibiotic extract. For bacterial cultures, samples were incubated for 18–24 h at 37 °C. The MIC dilution and at least two more concentrated dilutions were plated to count the number of CFUs to determine MBC (Kassim et al. 2016), state that, in comparison to the MIC dilution, the MBC is the lowest concentration that results in a 99.9% reduction in CFU/mL.

### Antibiofilm assay

The ability of the bioactive components from *Artemisia monosperma*, crude royal jelly, and their combination at ratio (1:1) to suppress biofilms was measured using 96-well polystyrene flat-bottom plates in a microtiter plate assay. and the final concentration of 10^^6^ CFU/mL was achieved by inoculating 300 μL of trypticase soy yeast broth (TSY) into each well. Then the sample was grown in the presence of previously determined sublethal doses (75%, 50%, and 25% of MBC). Controls are wells containing only medium and methanol. Plates were incubated at 37 °C for 48 h, and the resultant biofilm was stained for 15 min at room temperature with a 0.1% aqueous solution of crystal violet. The excess stain was eliminated by washing the plate three times with sterile distilled water. The dye attached to the cells was solubilized by adding 250 μL of 95% ethanol to each well, followed by a 15-min incubation, then the absorbance was measured using a microplate reader at a wavelength of 570 nm.

$$\begin{aligned}&{\text{Biofilm inhibition ability }}\% \,\\ &\quad\quad\quad\quad\quad = \, \frac{{{1} - \left( {{\text{Absorb sample }} - {\text{ absorb Blank}}} \right)}}{{\left( {{\text{Absorb}}.{\text{ Control}} - {\text{ absorb}}.{\text{ Blank}}} \right)}} \times 100\end{aligned} $$

### Anti-Alzheimer assay and IC_50_ values determination

Anti-Alzheimer activity of the bioactive compounds of crude *Artemisia monosperma*, fresh royal jelly and their (1:1) combination were measured for their ability to inhibit butyrylcholinesterase (BChE from Bio-diagnostic) using butyryl thiocholine iodide as substrates, based on a colorimetric method. Involving 10 μL of the test compounds solution in 0.2% DMSO, 79 μL of 20 mM sodium phosphate buffer (pH 7.6), and 1 μL enzyme preparation (with final concentrations: 0.2 µ/ml for BChE, and final concentrations: 0.195 to 100 μg/ml for compounds tested were mixed and preincubated for 15 min. To the mixture, 10 μL substrate solution was added (final concentration 4 mM for butyryl thiocholine iodide) and incubated for 30 min. The reaction was stopped by adding 900 μL DTNB-phosphate-ethanol reagent. The absorbance was measured at 405 nm using a microplate reader. The concentration of the test chemical substances required to inhibit BChE activity by 50% (IC_50_) was determined using an enzyme inhibition dose–response curve (Faisal et al. 2021).

### Molecular docking analysis of the *Artemisia monosperma* and fresh royal jelly bioactive compounds against Acetylcholinesterase protein

#### The bioactive components of *Artemisia monosperma* and crude royal jelly: Physicochemical, Drug-Likeness, and ADMET Properties

The physical and chemical characteristics of bioactive compounds obtained from PubChem database in SDF format (structure data format), involve drug-likeness attributes such as molecular weight, hydrogen bond donors, hydrogen bond acceptors, log *P* value, and rotatable bonds (Lipinski et al. 2012). According to (Zhang and Wilkinson 2007), compounds that follow Lipinski's rule of five should exhibit improved folding, polarity, and molecular size, which will result in better therapeutic effects. The ADMET webserver was employed to calculate the ADMET properties (Schyman et al. 2017) at http://www.swissadme.ch.

### Molecular docking

The three-dimensional structure of the Recombinant Human Acetylcholinesterase protein retrieved from protein data base (PDBID: 4EY7) with resolution: 2.35 Å (Cheung et al. 2012), the protein was prepared for docking using the Auto-Dock Tools 1.5.6 suite (Arumugam 2020). All non-protein atoms, including crystallographic waters and ligands, were removed. And the three-dimensional structure of *Artemisia monosperma* and crud royal jelly bioactive compounds Obtained from PubChem database in SDF (structure data format) (http://www.pubchem.ncbi.nlm.nih.gov), and prepared using Avogadro 1.2.0, (Arumugam 2020), in silico Molecular docking analysis were performed using Auto-Dock 4.2 (Swain et al. 2020). And the results were arranged based on the binding affinity. Finally, Protein (4EY7) and Ligand Complexes were visualized in 2D structure by BIOVIA Discovery Studio 2021.

### Statistical analysis

The data were analyzed using one-way analysis of variance (ANOVA; SPSS) to identify significant differences, represented as mean ± SD. The least significant difference multiple-range test was employed to analyze these differences (*p* ≤ 0.05).

## Results

### GC–MS analysis of the bioactive compounds in crud royal jelly and *Artemisia monosperma*

The crude bioactive compounds were evaluated by GC-Mass based on their chemical structures, molecular weights, and compound names, and the important compound was described in (Tables 1 and 2).

**Table 1:** Bioactive compounds of crud royal jelly by GC-Mass

No	RT	Compound name	Chemical formula	Molecular weight	Area%
1	16.78	3-(Tetradecyl-18O-oxy)-1,2-Propanediol	C_17_H_36_O_3_	288	2.16
2	20.00	9-Hexadecenoic acid	C_16_H_30_O_2_	254	3.29
3	20.19	Dodecanoic acid, 3-hydroxy-	C_12_H_24_O_3_,	216	3.44
4	21.21	17-Octadecynoic acid	C_18_H_32_O_2_	280	2.21
5	21.29	9-Octadecynoic acid(z)	C_18_H_34_O_2_	282	3.06
6	21.60	Aspidospermidin-17-ol,1-acetyl-16-methoxy-	C_23_H_30_N_2_O_5_	414	1.81
7	22.50	9,12,15-Octadecatrienoic acid,2-(acetyloxy)- [(acetyloxy)methyl] ethyl ester, (Z,Z,Z)-	C_25_H_40_O_6_	436	2.83
8	25.96	7-Methyl-Z-tetradecen-1-ol acetate	C_17_H_32_O_2_	268	1.86
9	26.40	Hexadecenoic acid 2,3-dihydroxypropyl	C_19_H_38_O_4_	330	9.49
10	28.64	6,9,12,15-Docosatetraenoic acid, methyl ester	C_23_H_38_O_2_	346	3.20
11	38.08	1-Heptatriacotanol	C_37_H_76_O	536	1.92
12	40.09	4H-1-Benzopyran-4-one,2-(3,4-dihydroxyphenyl)-6,8-di-á-D-glµcopyranosyl-5,7-dihydroxy-	C_27_H_30_O_16_	610	2.92
13	40.52	Loperamide	C_29_H_33_ClN_2_O_2_	476	4.13

Rt: Retention time

**Table 2:** Bioactive compounds of crude *Artemisia monosperma* by GC-Mass

NO	RT	Compound name	Chemical formula	Molecular weight	Area%
1	15.96	Methyl 6,8 octadecadienoate	C_19_H_30_O_2_	290	0.83
2	19.54	2-Naphthalenemethanol, decahydro-à,à,4a-trimethyl-8-methyle ne-, [2R-(2à,4aà,8aá)]-	C_15_H_26_O	222	6.19
3	19.73	10,12-Tricosadiynoic acid, methyl ester	C_24_H_40_O_2_	360	1.34
4	22.50	2-ACETYL-3-(2-Cinnamido) ethyl-7 Methoxy indole	C_22_H_22_N_2_O_3_	362	0.87
5	24.90	9,10-Secochola-5,7,10(19)-trien-24-a l, 3-hydroxy-, (3á,5Z,7E)-	C_24_H_36_O_2_	356	0.73
6	25.61	Cedrandiol, (8S,14)-	C_15_H_26_O_2_	238	0.99
7	26.35	Hexadecenoic acid,2,3-Dihydroxypropyl ester	C_19_H_38_O_4_	330	2.83
8	26.65	Valerenol	C_15_H_24_O	220	52.7
9	28.63	Androstan-17-one,3-ethyl-3-hydroxy-, (5à)	C_21_H_34_O_2_	318	1.83
10	28.74	Propanoic acid, 2-methyl-,(dodecahydro-6a-hydroxy-9a-methyl-3-methylene-2,9-dioxoazµleno[4,5-b]fµran-6-yl)methyl ester	C_19_H_26_O_6_	350	0.78
11	28.74	Retinol	C_20_H_30_O	286	0.78
12	29.03	1-Heptatriacotanol	C_37_H_76_O	536	1.24
13	30.05	9,10-SECOCHOLESTA-5,7,10(19)-TRIENE-1,3-DIOL,25-[(TRIMETHYLSILYL)OXY]	C_30_H_52_O_3_Si	488	1.21
14	30.94	9- OCTADECENOIC ACID (Z)-	C_18_H_34_O_2_	282	1.23
15	31.25	10,12,14-Nonacosatriynoic acid Glycidyl oleate	C_29_H_46_O_2_	426	0.90
16	34.30	Aristol-1(10)-en-9-ol	C_15_H_24_O	220	2.04

Rt: Retention time

### FTIR spectroscopy

**Fig. 2:**
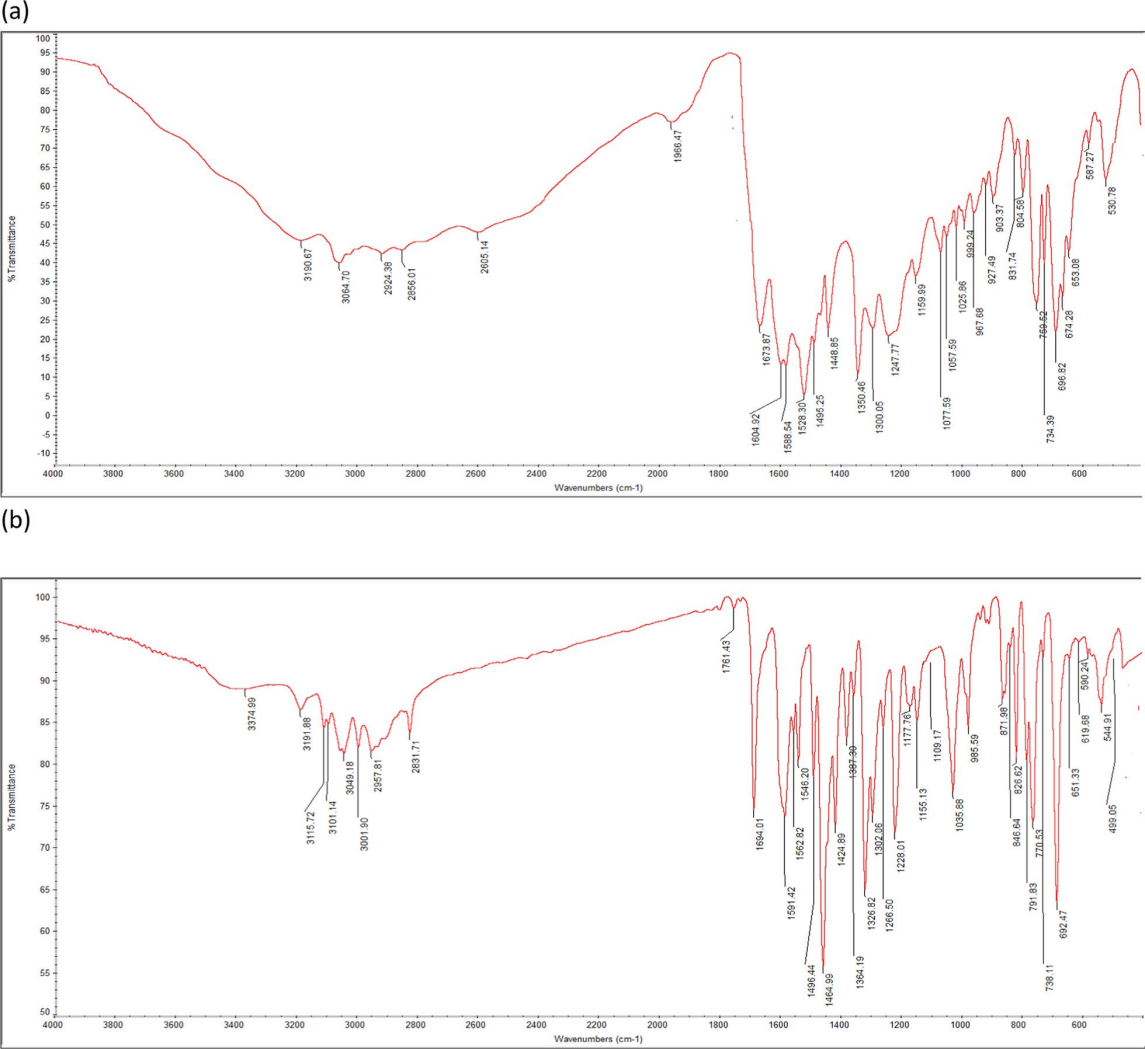
FTIR- analysis of **a ***Artemisia monosperma* leaves extract, **b** crud royal jelly

The presence of an aliphatic amine is confirmed by the band at 3190.67 cm^−1^, which is represented by N–H stretching, as shown in (Fig. 2a). While the C=N stretching seen at 2605.14 cm^−1^ demonstrated the existence of a nitrile functional group, the band at 2924.38 cm⁻^1^ suggests C–H stretching linked to alkane present. Furthermore, the band at 1673.87 cm⁻^1^ indicates the C=N stretching associated with conjugated imine or oxime molecules. The existence of an amine functional group is confirmed by the N–H bending at 1588.54. Alkanes are suggested by the C–H bending band at 1495.25 cm^−1^, whereas nitro compounds are suggested by the N–O stretching band at 1528.30 cm⁻^1^. Certain functional groupings are allocated the following wavenumbers: The corresponding carboxylic acid, sulfonate, aromatic ester, ester, tertiary alcohol, primary alcohol, alkene, and halo compound are 1448.85 cm^−1^ (O–H bending), 1350.46 cm^−1^ (S=O stretching), 1247.77 cm^−1^ (C–O stretching), 1159.99 cm^−1^ (C–O stretching), 1077.59 cm^−1^ (C–O stretching), 1057.59 cm^−1^ (C–O stretching), 967.68 cm^−1^ (C=C bending), and 696.82 cm^−1^ (C–I stretching), respectively. On the other hand, the primary functional groups in crud Royal Jelly are illustrated in (Fig. 2b), where a broad absorption band at 3191.88/cm is attributed to the –OH stretching mode. The band at 2957.81/cm corresponds to the C–C stretches of alkane linkages. The C=N stretching in amide groups is represented by the conspicuous absorption bands at 1694.01/cm. The prepared royal jelly's FTIR spectra shows an additional peak at 1035/cm in addition to the highlighted peaks. This peak, which is linked to the RCO-OH groups, shows that carboxylic acids are present.

### Antioxidant activity of *Artemisia monosperma* and royal jelly bioactive compounds

The results in Table S1 indicated that Ascorbic acid (standard control) showed the high DPPH scavenging% with the lowest IC_50_ as 2.53 ± 0.001 µg/ml followed by *Artemisia monosperma* leave extract with IC_50_ 5.48 ± 0.002 µg/ml, and Fresh royal jelly with IC_50_ 14.56 ± 0.002 µg/ml.

### Antibacterial activity of crude *Artemisia monosperma*, fresh royal jelly bioactive compounds and its composed.

**Fig. 3:**
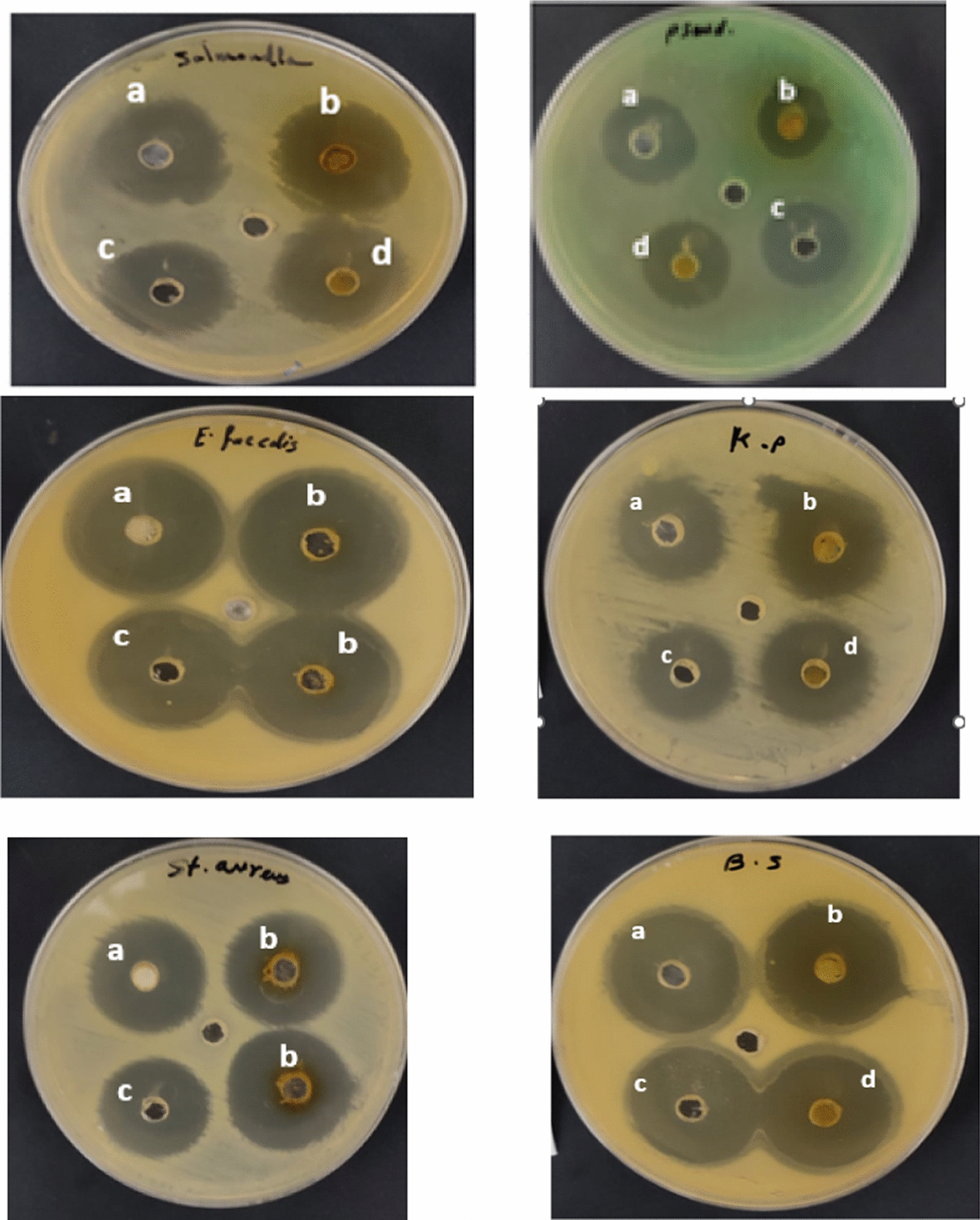
Antibacterial activity of **a** royal jelly **b**, *Artemisia monosperma* leave extract, **c**, gentamycin as control and **d**, composed (1:1)

Figure 3 shows antibacterial activities of *Artemisia monosperma* leaf extract, fresh royal jelly, and their (1:1) combination in compare with control (Gentamycin) against six bacterial strains as *Bacillus subtilis* (ATCC 6633), *Enterococcus faecalis* (ATCC 10541), *Staphylococcus aureus* (ATCC 6538), *K. pneumonia* (ATCC13883), *Salmonella typhi* (ATCC 6539) and *Pseudomonas aeruginosa* (ATCC 90274). The positive results measured as inhibition zone in (mm). *Artemisia monosperma* leaf extract being slightly mor effective in some case than fresh royal jelly but their (1:1) combination showed the highest antibacterial activity against pathogenic bacteria, particularly, *Enterococcus faecalis* (ATCC 10541) (32 ± 0.3 mm), *Staphylococcus aureus* (ATCC 6538) (33 ± 0.2 mm) So, the results suggest that the combination (1:1) of *Artemisia monosperma* leaf extract, fresh royal jelly could potentially more effective than either substance alone.

### MIC and MBC determination for *Artemisia monosperma* leaf extract, royal jelly and its composed (1:1)

**Fig. 4:**
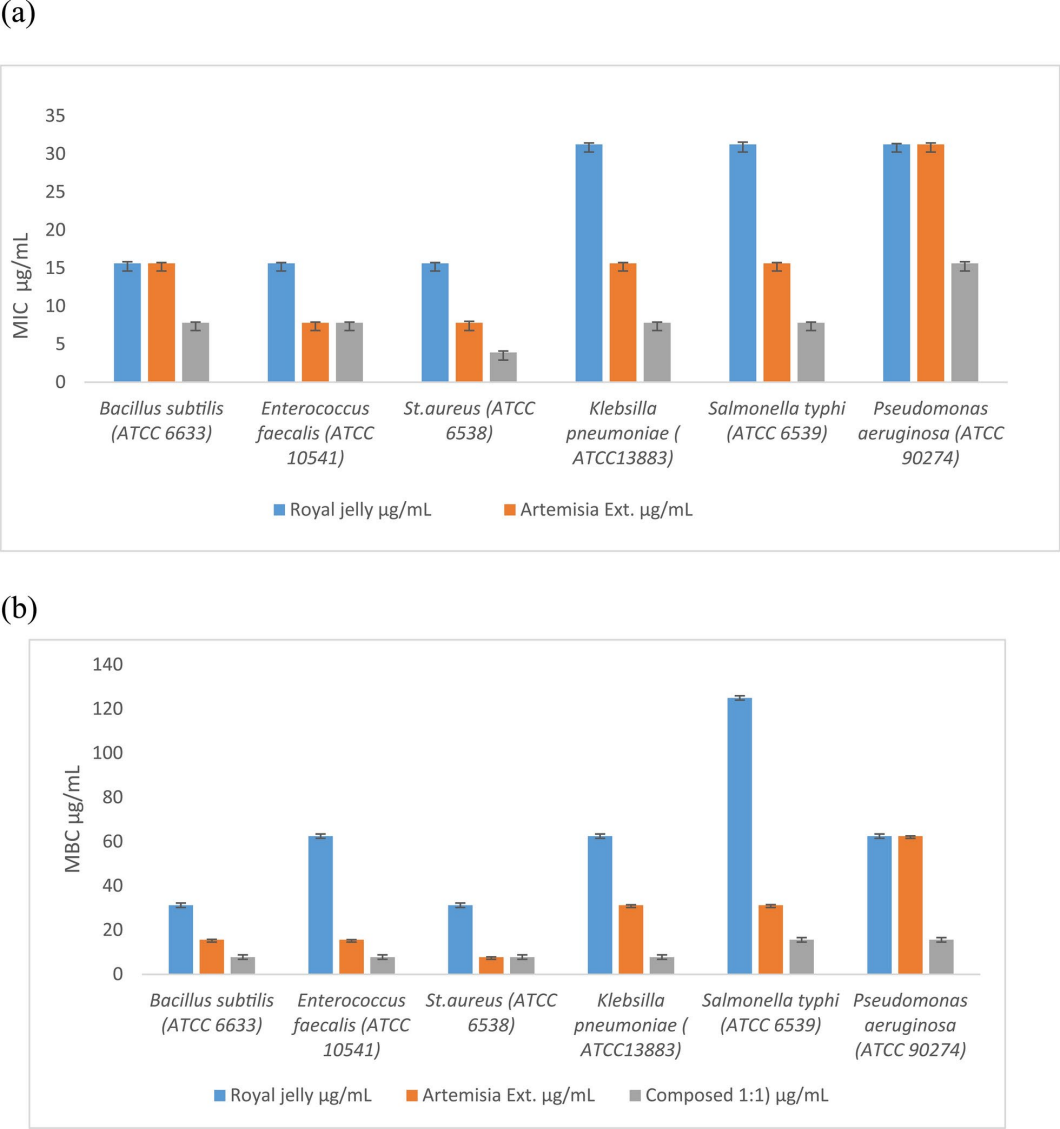
**a** The minimal inhibition concentration of *Artemisia monosperma* leaf extract, royal jelly and its composed (1:1) against pathogenic bacteria **b** The minimal bactericidal concentration of *Artemisia monosperma* and Royal Jelly and it’s composed (1:1) against pathogenic bacteria

Figure 4a shows the lowest concentration of antimicrobial agent (MIC) that completely inhibits growth of the pathogenic bacteria. The lowest concentration of royal jelly was 31.25 µg/mL and has ability to inhibit growth for, *Klebsilla pneumoniae* (ATCC13883), *Pseudomonas aeruginosa* (ATCC 90274) and *Salmonella typhi* (ATCC 6539), and *Enterococcus faecalis* (ATCC 10541), *Staph.aureus* (ATCC 6538) and *Bacillus subtilis* (ATCC 6633), growth at 15.62 µg/mL. And *Artemisia monosperma* leaf extract showed the lowest concentration as 31.25, 15.62, and 7.8 µg/mL that inhibit the growth of *Pseudomonas aeruginosa* (ATCC 90274, *Bacillus subtilis* (ATCC 6633) *K. pneumoniae* (ATCC13883) *Salmonella typhi* (ATCC 6539) *Enterococcus faecalis* (ATCC 10541), and *Staphylococcus aureus* (ATCC 6538) respectively. While the composed (1:1) of *Artemisia monosperma* leaf extract + royal jelly showed the best MIC value recorded as 3.9 µg/mL against *Staph.aureus* (ATCC 6538) than either substance alone.

On the other hand, the MBC minimal bactericidal concentration shown in Fig. 4b, is the lowest concentration that kill 99.9% in CFU/ml of bacterial population when compared to the MIC dilution, and the result showed in µg/mL. The lowest concentration of fresh royal jelly was 31.25 µg/mL as bactericidal against Bacillus *subtilis* (ATCC 6633) and *Staph.aureus* (ATCC 6538) while inhibit growth for *Enterococcus faecalis* (ATCC 10541), *Klebsilla pneumoniae* (ATCC13883) and *Pseudomonas aeruginosa* (ATCC 90274) at concentration 62.5 µg/mL in addition bactericidal activity against *Salmonella typhi* (ATCC 6539) at 125 µg/mL. In compare with of *Artemisia monosperma* leaf extract concentration 31.25, 15.62, and 7.8 µg/mL acting as bactericidal agent against *Salmonella typhi* (ATCC 6539), *Klebsilla pneumoniae* (ATCC13883), *Bacillus subtilis* (ATCC 6633), *Enterococcus faecalis* (ATCC 10541) and *Staphylococcus aureus* (ATCC 6538) respectively. While the composed (1:1) of *Artemisia monosperma* leaf extract and royal jelly showed the best MBC than either substance alone (Fig. 4b).

### Antibiofilm assay of *Artemisia monosperma* leaf extract, royal jelly and it’s composed (1:1)

**Fig. 5:**
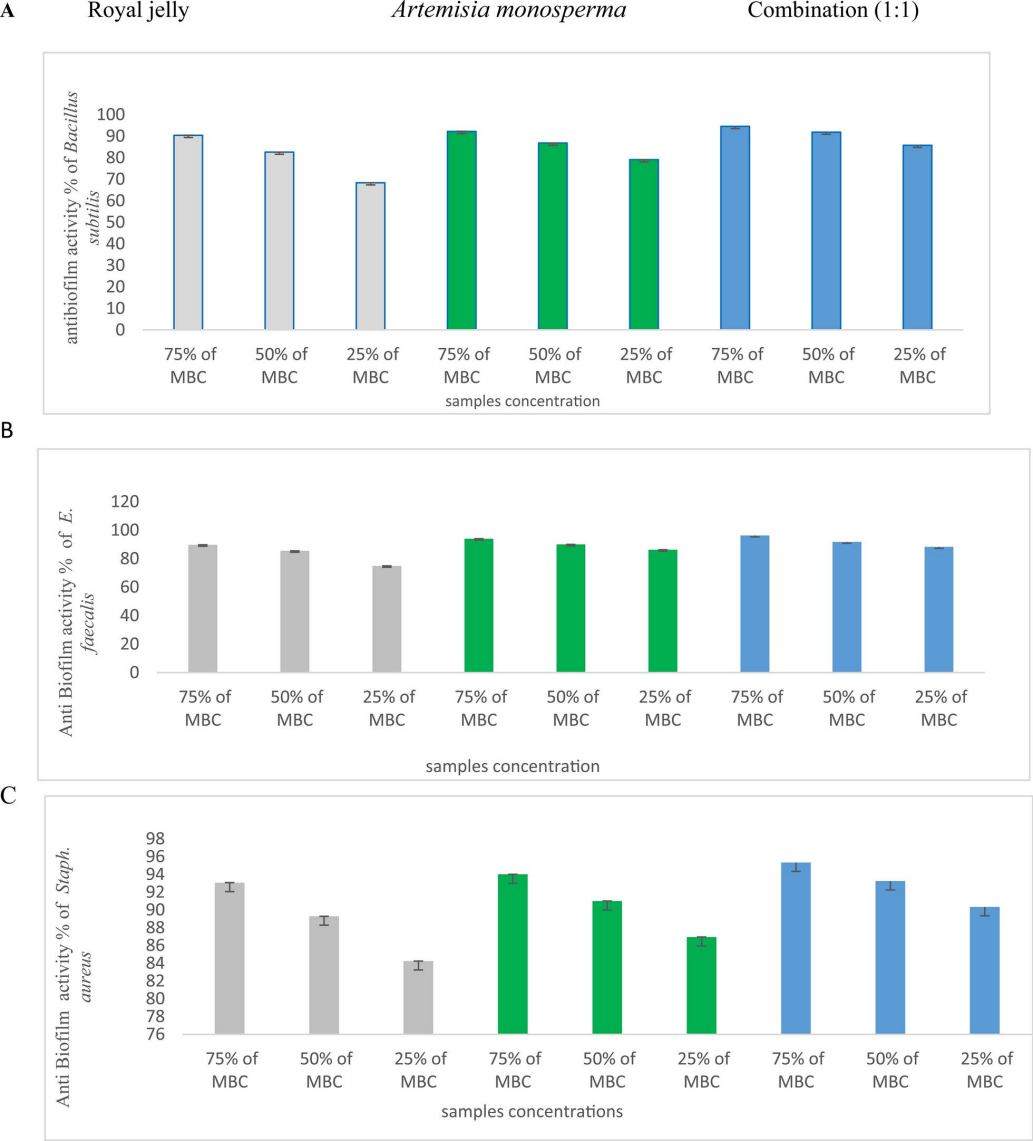
Anti-biofilm activity of *Artemisia monosperma* extract, crude royal jelly, and combination (1:1) against (**A**) *Bacillus subtilis*, (**B**) *Enterococcus faecalis* and (**C**) *Staph. Aureus*

**Fig. 6:**
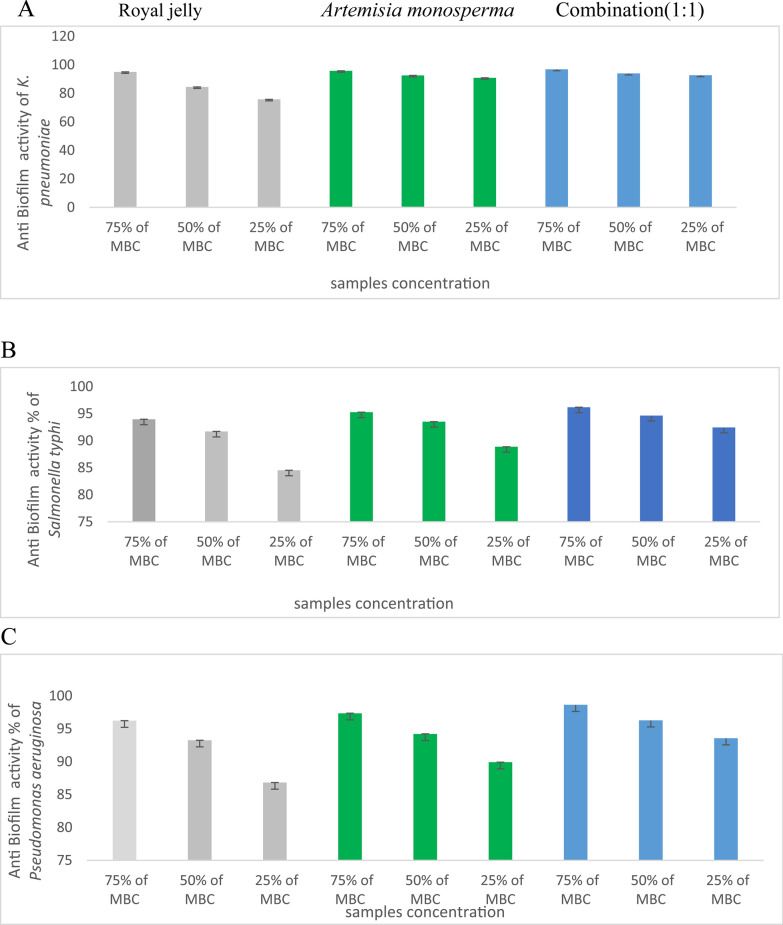
Anti-biofilm activity of *Artemisia monosperma* extract, crude royal jelly, and combination (1:1) against (**A**) *K. pneumonia*, (**B**) *Salmonella typhi* and (**C**) *Pseudomonas aeruginosa*

*Artemisia monosperma* leave extract, royal jelly showed the reduced biofilm of various bacteria viz., *Bacillus subtilis* (ATCC 6633) *and Staphylococcus aureus* (ATCC 6538)*, Enterococcus faecalis* (ATCC 10541), *K. pneumoniae* (ATCC13883)*, Pseudomonas aeruginosa* (ATCC 90274) and *Salmonella typhi* (ATCC 6539), compared with the composed (1:1) (Figs. 5, 6). So, *Artemisia monosperma* crude leaves extract, fresh royal jelly and its composed plays a role in the redaction of pathogenic bacterial biofilm.

### Anti-Alzheimer assay of *Artemisia monosperma* crude extract, fresh royal jelly, and its composed (1:1)

The findings regarding butyrylcholinesterase (BChE) indicated that both the *Artemisia monosperma* extract and crud royal jelly, as well as their 1:1 composition, effectively inhibited this enzyme in vitro, with IC50 values of 4.35 ± 0.002 µg/ml (*Artemisia monosperma extract)*, 4.9 ± 0.002 µg/ml (crud royal jelly), and 3.55 ± 0.002 µg/ml (1:1 composition), while the positive control Rivastigmine demonstrated an IC_50_ of 3.9 ± 0.002 µg/ml against BChE, as reported in Table S2.

### Molecular docking analysis of the *Artemisia monosperma* and fresh royal jelly bioactive compounds against Acetylcholinesterase protein

**Fig. 7:**
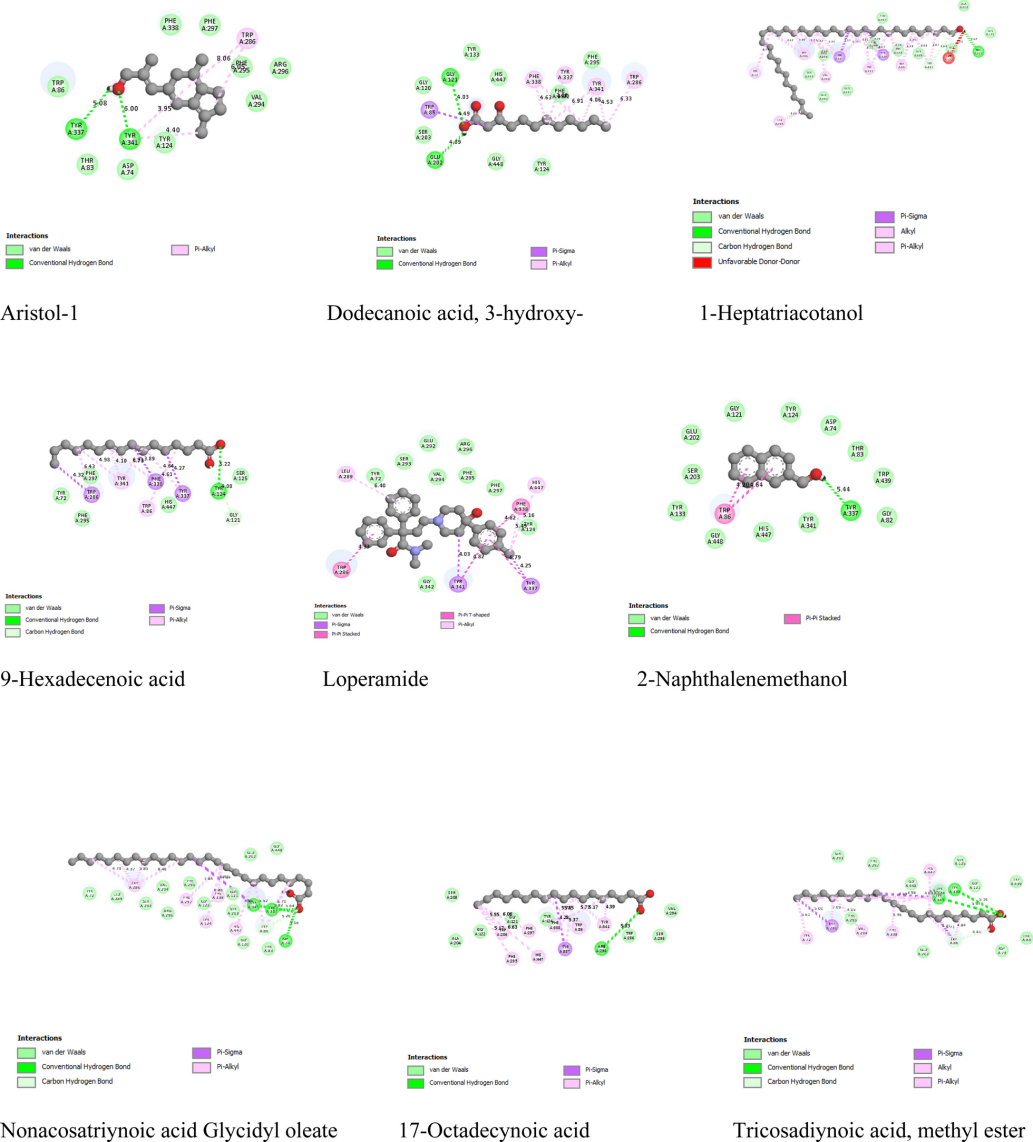
Molecular docking interactions between bioactive compounds from *Artemisia monosperma* and crud royal jelly with standard drugs against human Acetylcholinesterase protein

Nine bioactive compounds from *Artemisia monosperma* and crude royal jelly reach all five of Lipinski’s drug-likeness criteria, including a molecular weight of ≤ 500, a log P of ≤ 4.15, and the presence of N or O atoms ≤ 10. Pharmacokinetics indicated elevated gastrointestinal absorption and inhibition of CYP1A2 and CYP2C9. The results show that these compounds are attractive candidates for drug development and may serve as effective therapeutic agents for various conditions, including neurodegenerative diseases. larger potency is indicated by a larger negative docking score, which is always displayed as a negative number. Nine bioactive substances from fresh royal jelly and *Artemisia monosperma* showed docking scores against the target protein that ranged from − 6.6 to − 10.1 kcal/mol. Loperamide had the strongest effectiveness against the human acetylcholinesterase protein with high binding energy value of − 10.3 kcal/mol. However, 3-hydroxy dodecanoic acid showed the least potency with a binding energy value of − 6.6 kcal/mol (Table S3 and Fig. 7).

## Discussion

In this study, the evaluation of *Artemisia monosperma* have several chemicals compounds, including 3-(Tetradecyl-18O-oxy)-1,2-Propanediol, 9-Hexadecenoic acid, and Dodecanoic acid, 3 in line with El Zalabani et al. (2017), they showed the chemical profile of *Artemisia monosperma* arial parts, so the existence of these molecules indicates possible uses in the pharmaceutical industry, considering their established bioactive characteristic. besides, royal jelly is a white or pale-yellow secretion characterized by a strong, distinctive smell and taste (Kocot et al. 2018). The royal jelly have a diverse chemical composition as Methyl 6,8-octadecadienoate, 2-Naphthalenemethanol, and Valerenol Significantly, Valerenol included a significant proportion of the extract, indicating its crucial function in the biological effectiveness of royal jelly like with Guo et al. (2021). The identification of fatty acids and peptides in royal jelly like with the study conducted by Yükünç (2020). Moreover, FTIR spectroscopy provided additional clarification on the functional groups that were present in *Artemisia monosperma* and crud Royal Jelly have a significant impact on the their bioactivity and the extract of Artemisia monosperma leaves exhibited antioxidant activity due to its possible bioactive constituents according to Mashraqi et al. (2024). The robust antioxidant properties of *Artemisia* species are ascribed to their polyphenolic components (Soliman et al. 2024). Also royal jelly comprises numerous bioactive compounds that enhance its antioxidant properties, such as phenolic acids, flavonoids, and peptides researched by Guo et al. (2021).

Furthermore, in this study, the antibacterial, anti-biofilm assay of *Artemisia monosperma* and royal jelly bioactive compounds showed high against *Bacillus subtilis* (ATCC 6633), *Enterococcus faecalis* (ATCC 10541), *Staph. Aureus* (ATCC 6538), *K. pneumonia* ( ATCC13883), *Salmonella typhi* (ATCC 6539) and *Pseudomonas aeruginosa* (ATCC 90274) in agreement with previous studies (Mashraqi et al. 2024), *Artemisia monosperma* leaf extract exhibited higher antibacterial efficacy in comparison to crud royal jelly, while, the synergetic combination (1:1), exhibited the most potent antibacterial effect against the pathogenic bacteria Specifically, they were highly effective against *Enterococcus faecalis* (ATCC 10541) and *Staphylococcus aureus* (ATCC 6538), resulting in inhibition zones measuring 32 ± 0.3 mm and 33 ± 0.2 mm, respectively in agreement with the study conducted by Hasan et al. (2021a). Research by (Guo et al. 2021)on the antimicrobial properties of royal jelly demonstrated that it and its main components were particularly effective against gram-positive bacteria. *Bacillus subtilis* and *Bacillus cereus*, both gram-positive bacteria, produce spores in the final stage of their development, which protect them from unfavorable environmental conditions. This study showed that the royal jelly was the most effective against gram-positive bacteria *Enterococcus faecalis* (ATCC 10541), *Staph.aureus* (ATCC 6538) like with the study conducted by (Maželienė et al. 2022) Furthermore, the prior research conducted by (Altuntas et al. 2020)demonstrated that the royal jelly possesses the capacity to suppress biofilm formation both in vivo and in vitro within the food sector. This demonstrates the possible significance of royal jelly in diminishing biofilm development and preventing infection. While the bioactive compounds derived from *Artemisia monosperma* exhibit potential in inhibiting biofilm formation so this findings suggested that both are promising candidates against biofilm formation by various pathogenic bacteria, as *Staphylococcus aureus* and *Pseudomonas aeruginosa*, according to (Hasan et al. 2021b).The current study reported that the crud royal jelly *and Artemisia monosperma* bioactive compounds exhibit potent efficacy against pathogenic bacteria and in the inhibition of biofilm formation. Additionally, Lipinski's criteria were employed to evaluate the drug-likeness of nine bioactive chemicals extracted from *Artemisia monosperma* and fresh royal jelly. The compounds exhibited considerable gastrointestinal absorption and the ability to inhibit CYP1A2 and CYP2C9 enzymes. The docking research demonstrated that the binding affinities for the human acetylcholinesterase protein varied from − 6.6 to − 10.3 kcal/mol, with Loperamide displaying the best binding efficacy at − 10.3 kcal/mol. The results showed that the bioactive compounds have a promising role for neurodegenerative diseases treatment, in agreement with the study conducted by (Gnanaraj et al. 2022), which emphasized in Silico Analysis of potential natural drug design, development, and therapy targeting Alzheimer's and Parkinson's diseases. Finally, the current investigation provided that the GC-Mass analysis of local wild *Artemisia monosperma* crude extract and fresh royal Jelly reveals several bioactive components have promising antibacterial, anti-biofilm and anti-Alzheimer efficiency and in-silico analysis showed and predicted the mode of action of these compounds and their possible clinical applications.

## Supplementary Information

Supplementary Material 1

## Data Availability

All data generated or analyzed will be available in the main paper.

## Abbreviations

GC–MS: Gas chromatography–mass spectrometry GC–MS
DPPH: 2,2-Diphenyl-1-picrylhydrazyl
ADMET: Absorption, distribution, metabolism, and excretion–toxicity in pharmacokinetics
CYP1A2: A member of the cytochrome P450 mixed-function oxidase system
CYP2C9: Enzyme breaks down (metabolizes) compounds including steroid hormones
