# Supplementary material for: Synergistic Artemisia monosperma with royal jelly: antibacterial, antioxidant, antibiofilm, and anti-Alzheimer assay
Source: AMB Express. 2025 Mar 13;15:45. doi: 10.1186/s13568-025-01838-0 (PMC11906961; doi:10.1186/s13568-025-01838-0)
Supplement: Supplementary file 1 — Supplementary Material 1 [file 13568_2025_1838_MOESM1_ESM.docx]

**Table S1.** DPPH scavenging % of *Artemisia monosperma* and crud Royal Jelly in compare with ascorbic acid

| **samples** | **DPPH scavenging%(IC_50_)** |
| --- | --- |
| *Artemisia monosperma* leaves extract | 5.48 ± 0.002 µg/ml |
| Fresh royal jelly | 14.56 ± 0.002 µg/ml |
| Ascorbic acid (standard control) | - 1. 0.001 µg/ml |

**Table S2.** Cholinesterase inhibition data of *Artemisia monosperma* extract, fresh royal jelly, and its composed (1:1)

| **Sample** | **BChE IC_50_ in µg/ml** |
| --- | --- |
| *Artemisia monosperma extract* | 4.35 ± 0.002 µg/ml |
| fresh royal jelly | 4.9±0.002 µg/ml |
| composed (1:1) | 3.55 ± 0.002 |
| Rivastigmine (standard control) | 3.9 ± 0.002 µg/ml |

**Table S3.** binding affinity (kcal/mol) of Nine bioactive compounds from *Artemisia monosperma* and crud Royal Jelly with standard drugs against human Acetylcholinesterase protein

| Compound name | Binding affinity (kcal/mol |
| --- | --- |
| 9-Hexadecenoic acid | -6.9 |
| Dodecanoic acid, 3-hydroxy- | -6.6 |
| 1-Heptatriacotanol | -6.8 |
| Loperamide | -10.3 |
| Tricosadiynoic acid, methyl ester | -6.9 |
| 2-Naphthalenemethanol | -7.8 |
| 17-Octadecynoic acid | -7.3 |
| Nonacosatriynoic acid Glycidyl oleate | -6.9 |
| Aristol-1 | -9.1 |
| Rivastigmine * | −6.4 |

* Standard drugs
